# Imaging myocardial ischemia: from emerging techniques to state-of-the-art

**DOI:** 10.1186/s41747-021-00211-7

**Published:** 2021-03-25

**Authors:** Akos Varga-Szemes, Pal Suranyi

**Affiliations:** Division of Cardiovascular Imaging, Department of Radiology and Radiological Science, Medical University of South Carolina, 25 Courtenay Dr, Charleston, SC 29414 USA

**Keywords:** Artificial intelligence, Myocardial ischemia, Myocardial infarction, Tomography (X-ray computed), Magnetic resonance imaging

## Abstract

The widespread clinical use of cardiovascular imaging inspires constant improvement in imaging technology and post-processing applications. Recent advances in hardware and software have brought about important developments in the assessment of myocardial ischemia such as the rapid evaluation of cardiac volumes and function, ability for detection of subtle myocardial changes, and the combination of anatomic and functional assessment of a coronary artery stenosis via a single modality, which was previously not possible in a noninvasive fashion. These milestones indicate the start of a new era, a paradigm shift that broadens the role of noninvasive imaging. The thematic series *Myocardial tissue characterization in ischemic heart disease* introduces a set of narrative review and original articles by world renowned authors demonstrating such novel advancements and the state-of-the-art techniques in cardiac imaging.

Our editorial introduces a thematic series titled *Myocardial tissue characterization in ischemic heart disease*. Thematic series are a concept that *European Radiology Experimental* has adopted to examine and highlight advances in various technical and clinical areas of radiology. While the current pandemic clearly has an ongoing impact on academic productivity causing delays on every level of the publication process, we are grateful to the excellent authors for their tremendous effort to deliver these high-quality articles even under such challenging circumstances. We have the pleasure to kick off our thematic series with a combination of multiple narrative reviews and an original research article.

Imaging myocardial ischemia is a vibrant and exciting field of radiology research and clinical care. We spent the last two decades pursuing this field and have witnessed the development and evolution of cardiovascular imaging technology. Over the years, we have seen advances that made modalities more gentle [1], safer [2], faster [3], more accurate [4], but most importantly, we are fortunate to live in an era when noninvasive imaging claims new roles that, until recently, only invasive techniques would have been able to fulfill. As a good example, computed tomography (CT)-based fractional flow reserve (FFR-CT) may be one of the most impactful developments of the recent years with direct effect on the management of patients with suspected myocardial ischemia. FFR-CT offers an estimated FFR, a surrogate marker of myocardial hypoperfusion, which previously was only obtainable via invasive intracoronary catheterization and pharmacological stress [5]. The implementation of computational fluid dynamics allows an FFR to be derived from a standard coronary CT angiography (CTA) acquisition. This obviates the need for invasive angiography in some cases and expands the purely anatomical assessment of CTA with an additional functional dimension improving specificity for assessing patients with suspected coronary artery disease. While FFR-CT is only one example, it illustrates how a novel image post-processing approach may become a real game changer that directly affects patient care and management of ischemic heart disease.

The foundation of this thematic series is built upon three narrative review articles that cover emerging and clinically established imaging technologies used for myocardial ischemia assessment featuring authors from multiple institutions across the globe.

The first narrative review, titled *Emerging methods for the characterization of ischemic heart disease: ultrafast Doppler angiography, micro-CT, photon-counting CT, novel MRI and PET techniques, and artificial intelligence* by Willemink et al. from Stanford University, USA, provides an overview of techniques still in development but with high translational potential [6]. There are multiple novelties in this review article, of which we would like to highlight here at least three in this editorial. First, Dr. Willemink, a photon-counting CT expert [7], provides an overview of the technical background and a summary of the latest development in the field, including the potential outlook of bringing electrocardiographic gating into the photon-counting CT realm, therefore allowing for high-resolution, low noise *in vivo* human cardiac imaging. Second, the potentials of human myocardial imaging at low (≤ 0.35) and ultra-high (7 Tesla) magnetic fields are discussed, featuring the most important benefits and shortcomings of these technologies, new contrast agents with optimal relaxivity profiles at the respective fields, and image examples from Dr. Litt, from the University of Pennsylvania. Finally, this article takes a bit of an unconventional turn by summarizing the current status of artificial intelligence applications in cardiac imaging. While this topic is not strictly related to imaging technology, the plethora of artificial intelligence-based developments in image post-processing have been changing the way we interpret information and what additional information we can derive from images.

The second review paper in this thematic series, titled *Computed tomography for myocardial characterization in ischemic heart disease: a state-of-the-art review*, is delivered by van Assen et al. from Emory University, Atlanta, USA [8]. This article focuses on myocardial tissue characterization by discussing two major CT approaches: myocardial perfusion imaging and myocardial viability imaging. CT myocardial perfusion imaging, similar to the above mentioned FFR-CT approach, complements the anatomical evaluation offered by standard CTA with a more functional evaluation assessing the hemodynamic significance of coronary artery stenoses. Unlike FFR-CT, perfusion imaging using CT requires an additional acquisition during stress, increasing the total intravenous contrast dose and radiation exposure. The various acquisitions, including single or dual energy, static and dynamic techniques, are detailed by the authors along with recommendations on image evaluation and potential protocol optimization for reducing radiation dose. Myocardial viability imaging, the second focus of this paper, provides relevant information related to infarct location, extent, and pattern. This technique is based on late iodine enhancement, similar to MRI-based late gadolinium enhancement. Current CT technology, however, has inferior contrast-to-noise ratio for infarct visualization compared to MRI. The authors suggest improved late iodine contrast visualization and quantification may be achieved with promising developments in hardware such as improved contrast by photon-counting detectors or with software via texture analysis.

Lastly, the third paper of this review series, *State of the art CMR for myocardial characterization in ischemic heart disease*, is presented by Emrich et al. from Mainz University, Germany [9]. Cardiac magnetic resonance (CMR) has gone through multiple major milestones over the past years that not only improved its image quality and efficiency, but also broadened its utility. Compressed sensing technology has been developed providing highly accelerated image acquisition in a fraction of the time previously needed for cine acquisitions [10]. Such fast imaging improves time efficiency and widens the range of patients who can comfortably perform standard cine imaging by shortening the length of breath-holds, and therefore provides more accurate and physiologically more realistic evaluation of cardiac volumes, global systolic function and regional myocardial contractility. In the realm of regional wall motion evaluation, a relatively recent post-processing tool is worth noting, namely, feature tracking-based strain assessment [11]. Contrary to its predecessors, such as tagging, feature tracking is able to calculate strain parameters using standard cine images, therefore delivering advanced analytics regarding myocardial contractility without the need for additional image acquisitions. Possibly the most exciting field in CMR deserving a mention in our editorial is parametric myocardial mapping [12]. T1 and T2 mapping have been around for decades with evidence of their superiority relative to T1-weighted and T2-weighted imaging. However, only the past few years have brought the necessary technological advances making human myocardial mapping clinically feasible and practical. Novel clinical imaging sequences allow for the acquisition of T1 and T2 maps in a single breath-hold, with the post-processing embedded in the scanner software performing the actual pixel-by-pixel curve fitting in-line. These scanners generate parametric maps real time, with colorful displays sent directly to the radiologist workstations, allowing direct measurement of the calculated intrinsic physical parameters in the comfort of the reading rooms at the click of a button without the need for any additional fancy software or hardware. Research techniques show additional promising advancements, such as whole heart T1 mapping and multicontrast parametric mapping.

We are also delighted to announce an original research paper, entitled *Image quality of late gadolinium enhancement in cardiac magnetic resonance with different doses of contrast material in patients with chronic myocardial infarction*, submitted by a group from Milan under this thematic series at *European Radiology Experimental* [13]. In this work, Monti et al. investigated the optimal contrast dose for late gadolinium enhancement imaging to achieve sufficient image quality in patients with chronic myocardial infarcts. Considering the recent concerns related to gadolinium administration [14], the use of the smallest dose possible that still provides diagnostic quality images, is of high interest. The authors suggest that the administration of 0.15 mmol/kg gadobutrol for late gadolinium enhancement imaging provides significantly superior image quality when compared to the low dose of 0.10 mmol/kg, while image quality is similar to that obtained by administration of 0.20 mmol/kg.

In conclusion, the articles in this thematic series demonstrate the growing potential of noninvasive techniques that drive imaging of myocardial ischemia into a direction of fast and quantitative assessment of the myocardium. While it would be too early to predict the future potential of new imaging technology, there is a clear trend towards making cardiac imaging feasible on platforms that were previously unimaginable, such as ultra-high MR fields and spectral CT. These techniques may represent the next milestones in noninvasive imaging where we have the potential to reach substantially improved spatial and temporal resolutions and image contrast, as well as establish currently unknown quantitative biomarkers. Improvements to imaging techniques used in clinical standard of care are expected to gain further efficiency and become more gentle to patients by applying less radiation and contrast. The role artificial intelligence will play in the daily workflow of radiologists is not clear yet, however, developments are targeting both image assessment and image acquisition, by employing artificial intelligence-based algorithms to enhance and optimize patient-centered scan protocols.

Finally, we invite the radiology community to help grow this thematic series by submitting original research papers that provide us with the latest and greatest in advanced cardiac imaging and help keep this topical collection on our horizon.

## Data Availability

Not applicable.

## Abbreviations

CMR: Cardiovascular magnetic resonance
CT: Computed tomography
CTA: Computed tomography angiography
FFR: Fractional flow reserve
MRI: Magnetic resonance imaging
